# The Potential of HTS Approaches for Accurate Genotyping in Grapevine (*Vitis vinifera* L.)

**DOI:** 10.3390/genes11080917

**Published:** 2020-08-10

**Authors:** Urban Kunej, Aida Dervishi, Valérie Laucou, Jernej Jakše, Nataša Štajner

**Affiliations:** 1 Department of Agronomy, Biotechnical Faculty, University of Ljubljana, 1000 Ljubljana, Slovenia; urban.kunej@bf.uni-lj.si (U.K.); jernej.jakse@bf.uni-lj.si (J.J.); 2 Department of Biotechnology, Faculty of Natural Sciences, University of Tirana, Blv Zog I, 25/1, 1001 Tirana, Albania; aida.dervishi@fshn.edu.al; 3 AGAP, Univ Montpellier, CIRAD, INRAE, Montpellier SupAgro, F-34070 Montpellier, France; valerie.laucou@inrae.fr

**Keywords:** *Vitis vinifera* L., microsatellites, high-throughput sequencing, SSR markers, genotyping

## Abstract

The main challenge associated with genotyping based on conventional length polymorphisms is the cross-laboratory standardization of allele sizes. This step requires the inclusion of standards and manual sizing to avoid false results. Capillary electrophoresis (CE) approaches limit the information to the length polymorphism and do not allow the determination of a complete marker sequence. As an alternative, high-throughput sequencing (HTS) offers complete information regarding marker sequences and their flanking regions. In this work, we investigated the suitability of a semi-quantitative sequencing approach for microsatellite genotyping using Illumina paired-end technology. Twelve microsatellite loci that are well established for grapevine CE typing were analysed on 96 grapevine samples from six different countries. We redesigned primers to the length of the amplicon for short sequencing (~100 bp). The primer pair was flanked with a 10 bp overhang for the introduction of barcodes on both sides of the amplicon to enable high multiplexing. The highest data peaks were determined as simple sequence repeat (SSR) alleles and compared with the CE dataset based on 12 reference samples. The comparison showed that HTS SSR genotyping can successfully replace the CE system in further experiments. We believe that, with next-generation sequencing, genotyping can be improved in terms of its speed, accuracy, and price.

## 1. Introduction

Molecular marker technologies have changed plant genetics research enormously since their introduction in the 1980s and have provided researchers with a tool that is able to analyse an unlimited number of markers independent of environmental influences. Since then, there has been an impressive improvement in technology, which has moved from single to highly multiplexed analysis that includes southern hybridization probing [1]; random and specific PCR amplification methods [2,3]; quantitative PCR approaches [4]; microarrays [5]; and, more recently, next-generation (NGS) sequencing for plant genotype determination [6]. This progress has led to numerous publications describing dense genetic maps [7], finding quantitative trait loci (QTL) of great agronomic interest [8] and completely genotyped germplasm resources [9], to name a few.

The characterization of plant varieties or germplasm resources, such as grapevine, *Vitis vinifera* L., is a requirement driven by related economic interests, seed certification, plant variety rights, and scientific knowledge. Molecular marker methods for variety identification have undoubted advantages, including microsatellites, which have proven to be powerful tools for the identity, parentage, and kinship analysis of a wide range of plant species. Since their introduction in 1993 as a tool for plant genetic research [10], they have become one of the most widely used molecular markers in various fields of research, including plant genotyping. They are described as the best marker system for determining inter-variety polymorphisms [11]. Microsatellites, simple sequence repeats (SSRs) [12], or simple tandem repeat (STRs) [13] are the most commonly used DNA sequence features in plant genotyping due to their ubiquity in plants, their wide genomic distribution, their codominant inheritance, and their high degree of polymorphism [14,15]. Microsatellites are DNA regions consisting of tandem repeating units of 1–6 nucleotides. The number of repeats is highly variable between individuals due to the high rates of DNA polymerase slippage events [16] or unequal crossing-over [17], which makes them the ultimate multi-allelic marker system.

Microsatellite analysis is routinely based on multiplex fluorescence PCR, accompanied by capillary electrophoresis (CE) and the sizing of the resolved products. This is a fast and well-established technique with certain limitations: it is semi-quantitative, and the standardization of the identified alleles is required. When the CE methodology is applied between laboratories and the data subset is compared, the relative size values must be standardised against each other. In this step, manual sizing and processing are required, mainly due to the rounding of allele sizes, which must be very accurate to avoid false differences between samples from two data sets. The information provided by such an approach refers only to the length of the polymorphism and does not include the determination of the complete sequence of certain microsatellite loci.

Alternatively, new high-throughput sequencing platforms (HTS) enable the simultaneous sequencing of millions of sequences in a single run at enormous cost reductions [18]. The HTS analysis of microsatellite loci provides more information regarding SSR sequences, including the identification of sequence variants of STR loci that would be interesting for discriminating alleles, resolving mixed samples, and parentage analysis. Initial experiments successfully employed HTS platforms, such as Illumina and 454 sequencers for SSR genotyping, in the field of human forensic genetics, and showed the high applicability of powerful STR genotyping platforms [19,20]. Darby et al. [21] showed that such microsatellite genotyping is an ideal tool for population genetic structure studies, as it can detect a higher number of unique alleles compared to CE systems.

Recently, the term simple sequence repeats sequencing (SSRseq) was introduced to describe the application of HTS microsatellite genotyping. The authors developed a workflow for an efficient SSRseq setup for a wide range of situations [22]. In addition, the electrophoresis conditions associated with the polymer type [23], buffer conditions, or the use of alternative fluorescent dyes bound to primers [24] may also have an effect on DNA migration and the further sizing of microsatellite alleles. As denaturing electrophoresis resolves DNA fragments based on the length of the amplified alleles, fragments of equal length with different nucleotide compositions cannot be distinguished. This phenomenon is called size homoplasy [25], and can only be detected by sequencing the alleles.

In this work, the power of HTS for microsatellite genotyping was evaluated and a comparative genotyping study between HTS and a microsatellite CE analysis of grapevine cultivars was carried out. A standard set of 12 microsatellite loci was used to HTS-genotype 96 unique grapevine cultivars. In addition, a bioinformatic method is proposed using publicly available tools for sequence analysis. The microsatellite HTS analysis approach facilitates the high multiplexing capability of the loci and also allows the identification of variations that remain hidden in conventional SSR genotyping based on length polymorphisms.

## 2. Materials and Methods

### 2.1. SSRs and Cultivars

The genotyping of 96 grapevine cultivars (Table 1) obtained from six different countries (France, 12; Slovenia, 18; Bosnia and Herzegovina, 15; Serbia, 22; Montenegro, 5; Albania, 16; and North Macedonia, 8) was performed on 12 standard SSR loci using newly designed primers to shorten the product length below 150 bp (Table 2). The primers were designed using the Primer3 software [26]. A subset of HTS data was compared with the CE data of cultivars from the French collection (Table 1), obtained in a previous study of grapevine SSR genotyping, performed at National Research Institute for Agriculture, Food and Environment (INRAE), France [27].

### 2.2. DNA Extraction

The grapevine samples were obtained from different countries (Table 1), and DNA was extracted from fresh young leaves at the Biotechnical Faculty, University of Ljubljana, Slovenia. For this purpose, the modified cetyl trimethylammonium bromide (CTAB) method [28] was used. After measuring the concentrations (Amersham Biosciences DyNAQuant 200), the DNA samples were stored in a TE Buffer (Invitrogen™, Carlsbard, CA, USA) at −20 °C.

### 2.3. PCR Amplification

**Figure 1:**
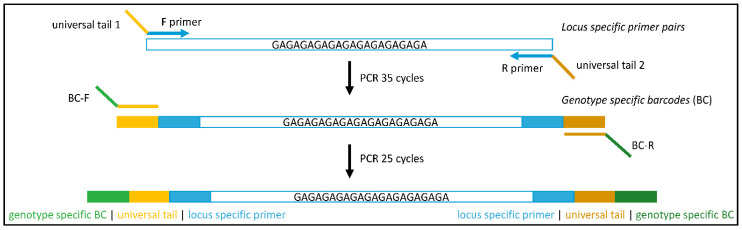
Workflow to amplify short sequence repeats in high-throughput sequencing (HTS) analysis. Amplifying begins with locus-specific amplification (step 1) using locus-specific forward (F) and reverse (R) primers extended with universal tails (Table 2); tail 1 (for F primer) is AATTAACCCT, tail 2 (for R primer) is CAGTCGGGCG. In step 2, the loci are pooled by sample and re-amplified to integrate the barcoding primers (BC-F, BC-R) listed in Supplementary Material, Figure S1.

The grapevine cultivars were genotyped using SSR amplicon sequencing. The primers were redesigned to the length of the amplicon for short sequencing (~100 bp) (Table 2) and amplified according to the established protocol. For this purpose, two rounds of PCR amplification were performed (Figure 1) according to the protocols of Gohl et al. [29] and Vartia et al. [30]. The modified protocol consisted of amplification with locus-specific primers (forward and reverse) adapted to contain a universal primer sequence (Figure 1; Table 3), and the incorporation of two barcodes by two barcoded universal primers into both ends of the resulting amplicons. A total of 12 forward and 8 reverse DNA barcodes enabled the recovery of 96 unique individuals (Supplementary Material, Figure S1).

#### 2.3.1. PCR for Locus-Specific Amplification

Primary PCR amplification was performed in a final volume of 10 µL containing 5 µL of 5X Q5 Hot Start HiFi buffer, 0.3 µL of 10 mM dNTPs, 5 µL of Q5 Enhancer, 0.1 µL of Q5 Hot Start HiFi Polymerase, 0.25 µL (10 µM) of each locus-specific primer (forward and reverse), and 20 ng of DNA. The cycling conditions were as follows: initial denaturation at 95 °C for 5 min, followed by 35 cycles of 98 °C for 10 s, 65 °C for 20 s, and 72 °C for 10 s. A final extension was performed at 72 °C for 2 min, and then the reaction was cooled down to 4 °C.

#### 2.3.2. PCR for Barcode Integration

We performed the second dual barcoding PCR in a volume of 10 μL containing 5 μL of primary PCR at a 1:100 dilution, 3 μL of 5 μM oligo for each index/barcode, 1.5 µL of 10x KAPA HiFi buffer, 0.3 µL of 10 mM dNTPs, and 0.08 µL of KAPA HiFi Polymerase. The following cycling conditions allowed the efficient incorporation of barcodes to PCR amplicons: initial denaturation at 95 °C for 5 min, followed by 25 cycles of 98 °C for 30 s, 45 °C for 30 s, and 72 °C for 1 min. A final extension was performed at 72 °C for 8 min, and the reaction was cooled down to 4 °C.

### 2.4. Pooling and Sequencing

After the second dual indexing PCR, the amplification products were checked using agarose gel electrophoresis across all loci and diluted appropriately to minimise the amplification rate differences between samples. Two microliters of each PCR product (across all loci and all specimens) were pooled together and cleaned using the Illustra GFX PCR and a gel band purification kit (GE Healthcare, Chicago, IL, USA), following the recommended procedures to remove shorter oligonucleotides. The cleaned sample was eluted in 25 µL, analysed with a highly accurate DNA electrophoresis Bioanalyzer 2100 system using a DNA 1000 kit (Agilent, Santa Clara, CA, USA), diluted to the final concentration of 20 ng/µL, and submitted for the Illumina 150 bp paired-end sequencing at GATC Biotech (Ebersberg, Germany). The project was designed to obtain approximately 5 M paired-end reads per DNA library. The reads were delivered as two FASTQ non-interleaved files.

### 2.5. Bioinformatics Analysis

Reference loci sequences were acquired through the Grape genome browser (12X coverage) (http://www.cns.fr/externe/GenomeBrowser/Vitis/) and adapted to shorter lengths (Table 2). The raw sequencing reads were mapped to the reference sequences using the “Map Reads to reference” tool implemented in CLC Genomics Workbench 20 (Version 20.0.3) (Qiagen, Hilden, Germany) to obtain the sequencing statistics per locus.

We used two different approaches to assign amplicon sequences to each cultivar and locus. The first approach consisted of mapping the raw sequencing data against the Pinot Noir genomic reference sequences. In the second approach, we demultiplexed the sequencing data by the cultivar- and locus-specific sequences present in the amplicon sequences. Briefly, the pair-end sequencing data were demultiplexed in two steps using the fastq-multx tool [34]. In the first step, the sequencing reads were demultiplexed based on the cultivar-specific barcodes introduced into amplicons in the second PCR step and, thus, sorted into the corresponding cultivar samples. After this, Cutadapt ver. 1.18 [35] was used to trim the cultivar-specific barcode sequences from the 3′ and 5′ ends of the reads.

In the second step, demultiplexing based on primer sequences, which are considered as locus-specific barcode sequences, was performed for each cultivar, and reads with locus-specific sequences on both ends of the reads were kept, thus retaining only full-length sequences. With this procedure, we filtered out incomplete amplicons and kept the reads that fully defined the microsatellite region. The filtered FASTQ files were converted to FASTA files and analysed using (1) the MISA Perl script [36] for the presence of perfect as well as compound microsatellites and (2) the Infoseq tool [37] to obtain the number of sequences with the same length.

The results were analysed with bash tools using the following procedure. The sizes of the microsatellites (no. of repeats or length of alleles) were reported for each read or amplicon sequence, and the number of unique values (sizes) were reported in a table-wise manner. The number of sequencing reads with obtained SSR sizes (MISA output) and the number of sequencing reads with obtained lengths (Infoseq output) were further used as an input for SONiCS [38], a tool that enables stutter noise correction and the determination of true alleles. The tool was run in Monte Carlo mode, with 5000 simulation repetitions. Analyses with SONiCS were applied for only a subset of data (12 French cultivars), for which we were able to make a comparison on the previously reported CE data [27].

## 3. Results and Discussion

### 3.1. Sequencing Analysis

The Illumina paired-end sequencing yielded 24,360,664 reads with an average size of 151 nt, yielding a total of 3,678,460,264 (3.68 Gb) bp of data. Theoretically, the even distribution over 12 loci should be approximately 306.5 Mb. The mapping of the reads to the reference alleles (Table 4) showed that the majority of the reads were of high quality, as 22 M of reads (90.7%) were assigned to 12 loci. However, the distribution of the reads across the loci was not uniform, with an acceptable range between 0.79 M for locus VVIq52 and 3.6 M for locus VMC1b11. This is most likely the consequence of competition among loci in the PCR during the library preparation.

The approach of using reference microsatellite sequences and further demultiplexing sequences based on mapping results did not prove to be the method of choice in our example. Microsatellite repeats can be similar between loci, which leads to incorrect mapping, especially for long alleles. Therefore, we chose a demultiplexing approach based on filtering out those sequences that contained correct locus-specific primer-to-primer information and were considered for the final genotyping. The final number of obtained reads was slightly lower than the number of mapped reads (19.4 M, 79.8%); however, they represented high-quality data that were confirmed twice by sequencing (the paired-end approach). Similarly, the demultiplexing approach yielded from 0.7 M (VVIq52) to 2.9 M (VMC1b11) full-length amplicons per locus (Table 4). Using the mapping approach, we obtained a slightly higher number of sequences for most loci; this was likely mainly due to the inclusion of sequences that did not cover the entire microsatellite sequences.

The minimum length of the amplicons demultiplexed by the locus ranged from 73 nt (VVIq 52 and VVIv37) to 99 nt (VVMD25), and the maximum length ranged from 85 nt (VVIq52) to 131 nt (VVMD25), corresponding to the allele lengths shown in the Supplementary Material, Figure S2.

### 3.2. Comparison of CE and HTS Approaches

The results of the comparisons between the HTS and CE methods for microsatellite analyses are presented in the Supplementary Material, Table S1. In examining the HTS approach, the sequences were analysed according to the number of microsatellite repeats (MISA script) and the full lengths of the sequenced amplicons (Infoseq script). The SSR lengths obtained by the MISA script and the amplicon lengths obtained by Infoseq were first analysed with SONiCS. During the visual inspection of the results, we found some allele calling errors when using automated SONiCS analyses, and thus we concluded that the approach using solely SONiCS was not appropriate for the determination of true alleles.

In the past, some other bioinformatics tools have been developed for retrieving SSRs from HTS data, such as LobSTR [39], RepeatSeq [40], STRViper [41], STR-FM [42], PSR [43], rAmpSeq [44], and STRScan [45]. We decided to use the software SONiCS, as it performs simulations of PCR reactions to correct allele calling due to the stutter bands, which are amplified at most grapevine SSR loci used in this study. SONiCS uses the length and depth of the sequenced alleles as input data, and, after each simulation, the log likelihood is calculated to estimate the probability of generating the observed data (input data) from the assumed simulated results. SONiCS selects the alleles for which the model has the highest likelihood. In 144 comparisons (12 loci × 12 cultivars) between MISA or Infoseq and the CE approach, SONiCS showed a 58% success rate in genotyping using MISA data, as 75 alleles were correctly called and 8 alleles differed only by 1 bp. When calling genotypes based on sequence length (Infoseq), SONiCS performed better compared to the approach using the MISA data, as it showed a 77% success rate in genotyping, as 102 alleles were correctly called and 9 alleles differed only by 1 bp.

However, due to missing some longer alleles with lower read counts, we continued to call alleles from the Infoseq output data by visual determination. The CE approach served as a standard. The comparison of the differences for the two alleles (per locus per sample) revealed some discrepancies between the HTS and CE methods, as shown in the Supplementary Material, Table S1. When comparing the MISA data with the CE data for 144 data points (12 loci × 12 cultivars), we obtained 75 alleles that showed the same difference between the alleles within the locus and 8 that differed only by 1 bp. Comparing the Infoseq data with the CE data for 144 data points, we obtained 102 alleles that showed the same difference between the alleles within the locus and 9 that differed only by 1 bp. The reported differences could be due to the development of new primers for HTS analyses that could lead to new null alleles, so that, in some cases, the homozygosity was higher than the expected heterozygosity for the HTS approach (Richter110, locus VVMD25), and, conversely, in some cases the homozygosity was higher than the expected heterozygosity for the CE approach (e.g., Merlot, locus VrZAG79).

**Figure 2:**
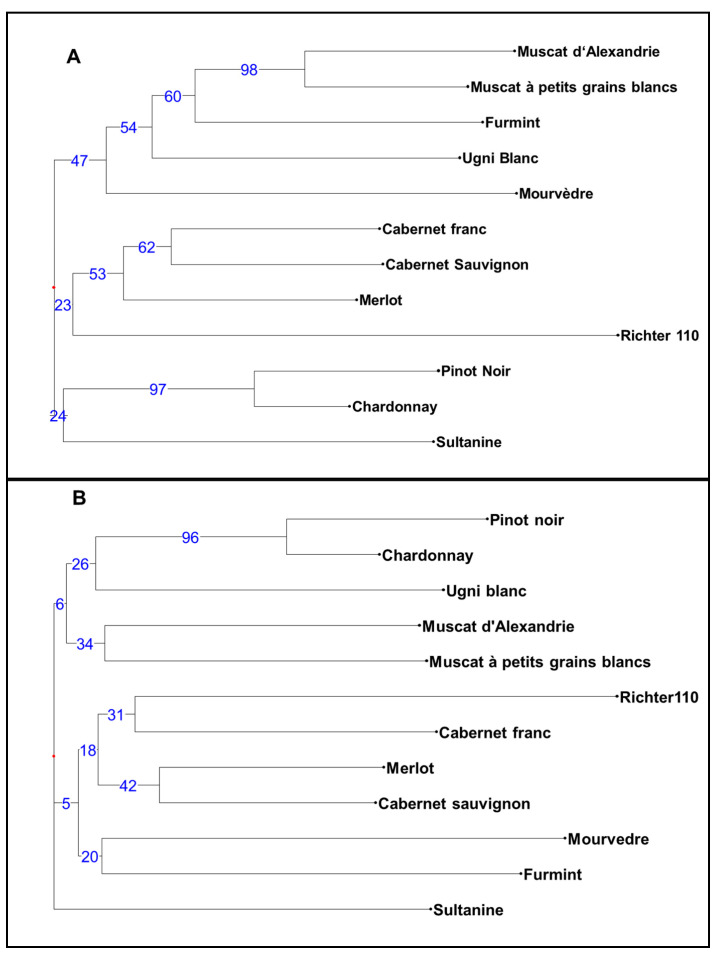
Tree construction based on simple-matching dissimilarity coefficient and the weighted neighbour-joining clustering method using alleles (**A**) obtained by capillary electrophoresis (CE) analysis (**B**) and by HTS (Infoseq) analysis. The numbers on the branches indicate the percentage of bootstrap analysis (1000).

The clustering of cultivars based on simple-matching dissimilarity coefficients was performed for the CE and HTS allelic data and resulted in two trees (Figure 2), with bipartition complexities of 0.94 and 0.91. The value for the consensus tree was 0.52, and the obtained distance between the trees was 0.82. Certain clusters supported with high bootstrapping values (e.g., a cluster of Muscat cultivars and cluster of Pinot Noir–Chardonnay) appeared equally in both approaches, and the Richter 110 rootstock was the most different from other *V. vinifera* cultivars in both approaches (Figure 2).

### 3.3. The HTS Approach Creates a Bias in Calling True Alleles for Some Loci

**Figure 3:**
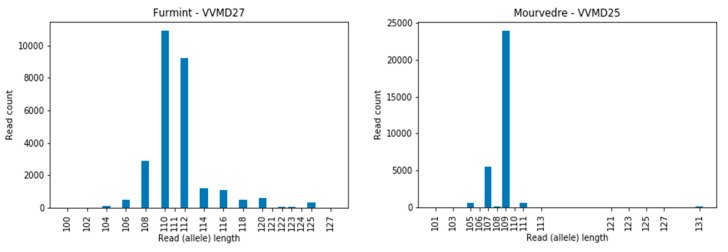
Example of the low sequence coverage for long alleles in the cultivar Furmint at locus VVMD27, allele 125 bp, and in cultivar Mourverde at locus VVMD25, allele 131 bp.

The number of read counts of full-length sequences (alleles) for 12 cultivars over 12 loci are presented as histograms (Supplementary material, Figure S2), with the corresponding alleles determined (Supplementary Material, Table S1; columns K and L). We observed that some loci are more problematic for the HTS approach than others; e.g., for the loci VVIq52, VVIb01, and VVMD24, we did not observe any discrepancies in the intra-allelic length comparison between different approaches (Supplementary Material, Table S1), whereas for locus VVMD27, for example, 6 out of 12 comparisons resulted in inconsistencies (Supplementary Material, Table S1). In locus VVIb01, the alleles were short (from 87 to 97 bp), and were similarly so in locus VVIq52 (from 75 to 83 bp) and VVMD 24 (from 97 to 108 bp), while in locus VVMD27 the allele lengths were from 110 to 125 bp and certain long-sized alleles could be overlooked due to their poor sequence coverage (Figure 3, Furmint, allele 125 bp). A similar problem was observed for the locus VVMD25 (Figure 3, Mourverde, allele 131 bp).

**Figure 4:**
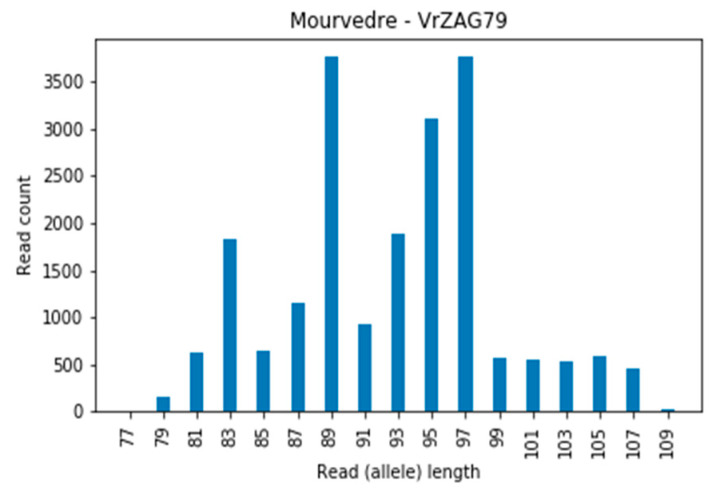
Example of the triallelic profile of cultivar Mourverde at locus VrZag79.

In locus VrZag79, in many cases (for cultivars Muscat Blanc a Petits Grains, Muscat d’Alexandrie, Mourvedre, Furmint, Cabernet franc, etc.) a three-allelic profile or high debris (reads of 83 and 89 bp) appeared. Figure 4 shows the Mourverde cultivar for locus VrZag79 with a tri-allelic profile (83, 89, and 97 bp). The three-allelic profiles discovered for this locus were previously observed in studies when extracting DNA from leaves. The presence of a third allele in leaf tissue indicates a periclinal chimera [46].

**Figure 5:**
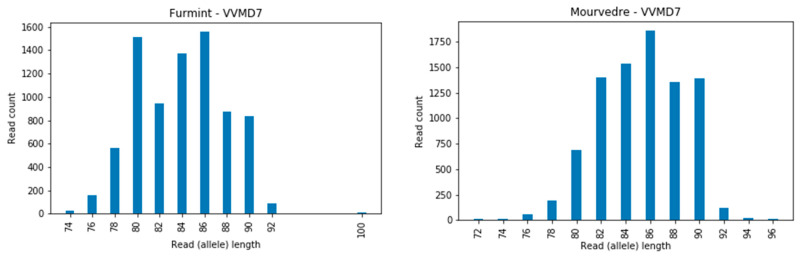
Example of the intense amplification of stutter bands at locus VVMD7 for two cultivars, Furmint and Mourverde.

The locus VVMD7 showed, in some cases, a very intensive amplification of stuttering bands (Figure 5), which can hinder the calling of true alleles. Small and unexpected mutations associated with locus VVMD7 were also reported earlier [46,47,48,49,50] and may, in some cases, be a consequence of the impaired allele calling.

### 3.4. Analyses of 96 V. vinifera Samples

The sequencing analyses (i.e., the number of reads for the sequenced amplicons) for 96 different *V. vinifera* cultivars over 12 loci are presented in the Supplementary Material, Table S2. In the analysed data set, we included five counterparts from French and Slovenian collections (Chardonnay, Merlot, Pinot Noir, Cabernet Sauvignon, and Sultanine), and the comparison over 12 loci yielded 55 exact matches and 5 discrepancies (Supplementary data, Table S2); three out of five were different for only two bp for the compared alleles and two were within the locus VVMD27, which was previously confirmed as one of the loci with triallelic profiles (chimerism) that showed a high intra-clonal variability [51,52]. Discrepancies were found in the Merlot and Pinot Noir cultivars, with previously reported intra-clonal genetic variation [46,51,52]. Studies have previously reported polymorphisms identified by microsatellite markers, which indicate the presence of trialellic loci, referred to in grapevines as chimeras [46,49], caused by mutations in the cells of the meristem layers L1 and L2 [53].

### 3.5. HTS Genotyping Economy

HTS systems offer extremely cost-effective sequencing generation for large amounts of data. Therefore, HTS systems are already used in genotyping projects that employ different strategies to find polymorphisms, such as genotyping by sequencing [54], capturing strategies [55,56,57], or the shotgun sequencing of entire genomes [58]. Microsatellites are multiallelic markers, which makes them ideal for the management of plant germplasm. In our project, we investigated the possibility of using a sequence counting approach for genotyping microsatellite alleles.

There are also economic reasons behind switching from capillary-based systems to HTS platforms. The first important reason is the price of a capillary-based instrument, which is higher than for medium-throughput NGS systems. The price of the instrument is worth considering, especially for those laboratories that are considering either replacing their capillary systems or buying new ones. The second reason is the operating costs. The sequencing cost of our project was 531 € (VAT excluded), and we have produced more than 12 million sequences. Our data contained 1152 data points (96 cultivars by 12 loci), which means 0.46 € per data point. However, the sequencing coverage was extremely high (10,000× on average). We believe that we were able to reduce the coverage by at least five times, which is 0.09 € per data point. The running costs for capillary instruments are higher than 1 € per sample (data point), and genotyping providers usually charge 2.5–3 € per sample. Therefore, the economic situation speaks in favour of HTS typing.

## 4. Conclusions

The remarkable advances in high-throughput sequencing technologies have significantly increased their application in genetic diversity studies, population structure analyses, and conservation genetics. The HTS approach has the advantage of the large-scale genotyping of individuals at multiple loci simultaneously using an amplicon barcoding system that allows large-scale analysis, generating a large amount of data in less time and at a surprisingly lower cost [59,60]. The HTS approach showed significant advantages over the fragment length variation-based approach using conventional capillary and gel electrophoresis [21,30,59,61]. Studies [21,59] reported that HTS technology increased the number of detected alleles compared to the electrophoresis-based method, overcoming the effect of microsatellite length homoplasy, resolving the hidden variations, and maximizing the genetic information obtained. While homoplasy was reported in certain previous studies, it was not detected in any of the loci we investigated. Homoplasy is more likely to be detected in less closely related genotypes.

According to our observations, the limitation of HTS-SSR genotyping is in the automation of allele retrieval, which is crucial for HTS approaches with high multiplexing and large amounts of data. Due to the high degree of mismatching observed for some microsatellite loci when using SONiCS bioinformatics tools for retrieving SSRs from HTS data, we recommend that other tools should be investigated and/or improvements made to the existing tool (e.g., the normalization of the read counts according to the amplicon length and sequencing depth of the libraries) to reduce the distortion obtained from the amplification and sequencing process.

The HTS-SSR approach has huge potential in terms of its speed and cost effectiveness. As our study is one of the first studies of this kind presented for plants, an additional optimization and validation process should be performed before the routine use of HTS genotyping instead of the CE approach, especially as we have shown that not all loci are equally suitable for the sequencing approach.

## Figures and Tables

**Table 1:** The 96 cultivars analysed in this study, sorted by barcodes and assigned to their country of origin.

Barcode	Cultivar	Country of Origin	Barcode	Cultivar	Country of Origin
F1-R1	Kratošija	North Macedonia	F7-R1	GnetKras	Slovenia
F1-R2	Neznana Bela	Slovenia	F7-R2	Kratošija I	Montenegro
F1-R3	Rebula	Slovenia	F7-R3	Muštoš Feher	Serbia
F1-R4	Sremska Zelenika	Serbia	F7-R4	Rebula Portalis	Slovenia
F1-R5	Zimsko Belo	Serbia	F7-R5	Smederevka	Bosnia and Herzegovina
F1-R6	Manastirsko Belo	North Macedonia	F7-R6	Vranac	Bosnia and Herzegovina
F1-R7	Dobrogostina	Bosnia and Herzegovina	F7-R7	Belovina	North Macedonia
F1-R8	Godominka	Serbia	F7-R8	Gnjet	Slovenia
F2-R1	Bagrina	Serbia	F8-R1	Kreaca	Serbia
F2-R2	DrenakCrni	Serbia	F8-R2	Refosco	Slovenia
F2-R3	Kadarka Bela	Serbia	F8-R3	Stanušina	North Macedonia
F2-R4	Krkošija Šupljica	Serbia	F8-R4	Žametovka	Bosnia and Herzegovina
F2-R5	Prokupac	Bosnia and Herzegovina	F8-R5	Ohridsko Belo	North Macedonia
F2-R6	Ružica	Serbia	F8-R6	Refošk	Slovenia
F2-R7	Bela Zgodnja	Slovenia	F8-R7	DolgiGrozdi	Slovenia
F2-R8	**Chardonnay**	Slovenia	F8-R8	Gročanka	Serbia
F3-R1	Drenak	Bosnia and Herzegovina	F9-R1	PlovdinaCrna	Serbia
F3-R2	Kadarka	Serbia	F9-R2	Rezaklija	Bosnia and Herzegovina
F3-R3	Kujundžuša	Bosnia and Herzegovina	F9-R3	Stari Rizling VI	Montenegro
F3-R4	Prokupac	Serbia	F9-R4	Žlozder	Bosnia and Herzegovina
F3-R5	Ružica V	Montenegro	F9-R5	Debinë e Zezë	Albania
F3-R6	TamjanikaCrna	Serbia	F9-R6	Kallmet	Albania
F3-R7	**Merlot**	Slovenia	F9-R7	Potek e Zezë	Albania
F3-R8	Beli Medenac	Serbia	F9-R8	Shesh i Zi	Albania
F4-R1	Ružica VI	Montenegro	F10-R1	Stambolleshë	Albania
F4-R2	TrbljanBeli	Serbia	F10-R2	Sheshi Bardhë	Albania
F4-R3	**Pinot noir**	Slovenia	F10-R3	Kosinjot	Albania
F4-R4	Bena	Bosnia and Herzegovina	F10-R4	Vlosh	Albania
F4-R5	Elezovka	Bosnia and Herzegovina	F10-R5	Tajgë e Zezë	Albania
F4-R6	Kavčina	Serbia	F10-R6	Meresnik	Albania
F4-R7	Marburger	Slovenia	F10-R7	Korith i Bardhë	Albania
F4-R8	Prošip	Bosnia and Herzegovina	F10-R8	Tajgë e Bardhë	Albania
F5-R1	Sipa	Slovenia	F11-R1	Pulëz	Albania
F5-R2	Trnjak	Bosnia and Herzegovina	F11-R2	Razaki e Kuqe	Albania
F5-R3	**Cabernet Sauvignon**	Slovenia	F11-R3	Serinë e Bardhë	Albania
F5-R4	BlatinaI	Bosnia and Herzegovina	F11-R4	Debinë e Bardhë	Albania
F5-R5	Furmint	Serbia	F11-R5	Furmint	France
F5-R6	Menigovka	Bosnia and Herzegovina	F11-R6	**Chardonnay**	France
F5-R7	Radovača VII	Montenegro	F11-R7	**Pinot Noir**	France
F5-R8	Sipon	Slovenia	F11-R8	Mourvedre	France
F6-R1	**Sultanine**	Slovenia	F12-R1	Ugni B/Trebbianotoscano	France
F6-R2	CrnValandovskiDrenok	North Macedonia	F12-R2	Muscat a petit grains	France
F6-R3	Gavran	Serbia	F12-R3	Muscat d’Alexandrie	France
F6-R4	Končanka	North Macedonia	F12-R4	**Merlot**	France
F6-R5	Muskat Ruža	Serbia	F12-R5	**Cabernet Sauvignon**	France
F6-R6	Slankamenka Crvena	Serbia	F12-R6	Cabernet franc	France
F6-R7	**Touriga Nacional**	Slovenia	F12-R7	**Sultanine**	France
F6-R8	Čauš Bel	North Macedonia	F12-R8	Richter110	France

* Bolded cultivars are, in general, considered as references.

**Table 2:** The reference sequence, microsatellite core repeat, and reference length of each locus.

Locus	Reference Sequence	Microsatellite Core Repeat	Reference Length
VMC1b11-NGS	GACCTAAGTTTCTGAGGCTTTGAAAATTACCTTCCGGGTTTCTAGAGAGGGAGAGAGAGAGAGAGAGAGAGAGAGAGGAAGGTTCGGCAACACAAAATGAGAGGCA	(GA)n	106
VrZAG79-NGS	TTAGCCGAAGCCATCTCTGTTCTCAAGCAGAATGGAAGTGAGAGAGAGAGAGAGAGARGAGAGAGAGAGAGATAAAGGTGGTGAGGTGCTTGTGTTTCTTGA	(CT)n	102
VVIb01-NGS	CCTGTGAAACCACCACTATCCTCAGAGAAGCTCTCTCTCTCTCTCTCTCTCTTCACACTCACATCACTCGTTTACCTTGTGCAACCA	(CT)n	87
VVIn73-NGS	AGGCTTCAAAGCCCTCTCATCTTAATTCGTGTGTGTGTGTGTGTGTGTTGGGGCCTTTGGGGCTCCACTGACACCCACAAGGGTGT	(CA)n	86
VVIp31-NGS	TTGGGAAACCACAGAAGTGACAATTTATAGAGAGAGAGAGAGAGAGAGAGAGAGAGAGAGGCATATCCATTAGAATGATCACATTCCAGGAACAACCCATT	(GA)n	101
VVIq52-NGS	CAGGAAAGTGTTCAATGGTTACAAAACAGGAGAGAGAGAGAGAGAGAGTGTGTCACTGGTTCTGTCATCTACCATCCTT	(CT)n	79
VVIv37-NGS	ACCAGTATTAAGAACGCAGTCACTGCCCACAGAGAGAGAGAGAGAGAGAGAGAGAGAGAGAGAGAGAGAGAGATGGGGTGAGTGGGAAGTTAAGAGTAGGG	(TC)n(GT)n	101
VVMD24-NGS	AGAAGACTTGTCTCTCTCAATCAAATTGTGGTCCTCCTCTCTCTCTCTCTCTCTCTCTCTCTACTACTGCATATCATTGATAGTCCTTGTCTCAATTTCTTTGCG	(CT)n	105
VVMD25-NGS	TGAAAAGTGTAGTGACCCTTTGACTAGGCCTCCCTTCTCTCTCTCTCTCTCTCTCATGTTTATGTTATTTATTGTTTTTTTCCTTGAAACCACAAGACAAGCCTCCA	(CT)n	107
VVMD27-NGS	CCTCTCTCTCCGGCGGTATTCTCAATCTCCCTCCTCCTTCCGCCCAAGTTGAGGTCTCTCTCTCTCTCTCTCTCTCTCTATTTATATACTTACGGATGTATTCAGATCTGGT	(CT)n	112
VVMD32-NGS	TGAAACGTCTCGCCATTACCCCTCCCTCTCTCTCTCTCTCTCTCTCTCTCTCTCTCTCTCTCTCTCTCTCTCTCTCTCAAGCCAGGCGTCAAAACATGAACTGTTTGTC	(CT)n	109
VVMD7-NGS	CCTCAAGCAGCGTATCCATAGCGAGTGGAGGAGAGAGAGAGAGAGAGAGAGAGAGAGAGAGTGAGCGCCAAAGAGAGAGGGAGGAGGG	(CT)n	88

**Table 3:** Table of the simple sequence repeat (SSR) locus-specific primers with universal tail (letters in bold), linkage group, and reference.

SSR Name	Linkage	Locus Specific Forward Primer with Universal Tail	Locus Specific Reverse Primer with Universal Tail	Reference
VMC1b11-NGS	8	**AATTAACCCT**GACCTAAGTTTCTGAGGCTTTGA	**CAGTCGGGCG**TGCCTCTCATTTTGTGTTGC	BV681754
VrZAG79-NGS	5	**AATTAACCCT**TTAGCCGAAGCCATCTCTGT	**CAGTCGGGCG**TCAAGAAACACAAGCACCTCA	[31]
VVIb01-NGS	2	**AATTAACCCT**CCTGTGAAACCACCACTATCC	**CAGTCGGGCG**TGGTTGCACAAGGTAAACGA	[32]
VVIn73-NGS	17	**AATTAACCCT**AGGCTTCAAAGCCCTCTCAT	**CAGTCGGGCG**ACACCCTTGTGGGTGTCAGT	[32]
VVIp31-NGS	19	**AATTAACCCT**TTGGGAAACCACAGAAGTGA	**CAGTCGGGCG**AATGGGTTGTTCCTGGAATG	[32]
VVIq52-NGS	9	**AATTAACCCT**CAGGAAAGTGTTCAATGGTTAC	**CAGTCGGGCG**AAGGATGGTAGATGACAGAACCA	[32]
VVIv37-NGS	10	**AATTAACCCT**ACCAGTATTAAGAACGCAGTCAC	**CAGTCGGGCG**CCCTACTCTTAACTTCCCACTCA	[32]
VVMD24-NGS	14	**AATTAACCCT**AGAAGACTTGTCTCTCTCAATCAAA	**CAGTCGGGCG**CGCAAAGAAATTGAGACAAGG	[33]
VVMD25-NGS	11	**AATTAACCCT**TGAAAAGTGTAGTGACCCTTTGA	**CAGTCGGGCG**TGGAGGCTTGTCTTGTGGTT	[33]
VVMD27-NGS	5	**AATTAACCCT**CCTCTCTCTCCGGCGGTA	**CAGTCGGGCG**ACCAGATCTGAATACATCCGTAA	[33]
VVMD32-NGS	4	**AATTAACCCT**TGAAACGTCTCGCCATTACC	**CAGTCGGGCGG**ACAAACAGTTCATGTTTTGACG	[33]
VVMD7-NGS	7	**AATTAACCCT**CCTCAAGCAGCGTATCCATAG	**CAGTCGGGCG**CCCTCCTCCCTCTCTCTTTG	[33]

**Table 4:** Sequencing statistics for 96 grapevine cultivars over 12 loci.

Locus	Reference Allele Length	Mapped Reads ^1^	Amount of Data [bp] after Mapping ^2^	Average Coverage after Mapping	No. of Sequences after Filtering ^3^	Amount of Data (bp) after Demultiplexing	Average Coverage after Filtering
VMC1b11	106	3,649,804	551,120,404	2,888,215	2,927,098	305,691,052	2,883,878
VrZAG79	102	1,192,832	180,117,632	1,148,842	1,694,202	156,444,090	1,533,766
VVIb01	87	2,713,933	409,803,883	2,712,447	2,444,143	223,916,517	2,573,753
VVIn73	86	1,085,003	163,835,453	1,084,374	957,723	81,434,445	946,912
VVIp31	101	1,963,722	296,522,022	1,819,017	1,447,397	149,040,267	1,475,646
VVIq52	79	792,078	119,603,778	791,626	704,921	56,007,493	708,956
VVIv37	101	1,119,484	169,042,084	1,022,452	1,264,019	115,785,796	1,146,394
VVMD24	105	1,813,906	273,899,806	1,810,404	1,588,685	162,085,558	1,543,672
VVMD25	107	2,348,776	354,665,176	2,192,870	2,000,291	221,586,577	2,070,903
VVMD27	112	2,911,241	439,597,391	2,895,057	2,468,380	277,195,183	2,474,957
VVMD32	109	1,556,828	235,081,028	1,163,432	1,037,861	92,983,922	853,064
VVMD7	88	949.509	143,375,859	937,902	899,408	75,335,819	856,089
	Total	22,097,116	3,336,664,516		19,434,128	1.917,506,719	

^1^ Raw reads mapped to reference alleles using CLC genomics Workbench/Server. ^2^ Total number reported with unaligned part included. ^3^ Full length sequences starting and ending with amplification primer.

## Supplementary Materials

The following are available online at https://www.mdpi.com/2073-4425/11/8/917/s1: Figure S1: (**a**) Forward and reverse barcodes with universal tail (in bold); (**b**) Barcoding system in a 96-well plate; 12 forward barcodes and 8 reverse barcodes enabling the barcoding of 96 samples. Figure S2: Histograms generated from the number of read counts of full-length sequences (alleles) obtained with the Infoseq approach for twelve *V. vinifera* cultivars at twelve different loci. Table S1: Comparison of three different approaches to determine the genotypes of 12 different *V. vinifera* cultivars at 12 different loci, i.e., by capillary electrophoresis (CE), by the calling length of SSR (MISA), and by the calling allele lengths (Infoseq). The genotype data obtained by capillary electrophoresis are publicly available [27]; SONiCS was used to call genotypes from the data obtained by MISA, and visual determination of genotypes was done to call the alleles obtained by Infoseq. Table S2: The No. of reads for the sequenced amplicons of 96 different *V. vinifera* cultivars of locus VMC1b11 obtained using the HTS approach. Table S2.1: The No. of reads for the sequenced amplicons of 96 different *V. vinifera* cultivars of locus VrZAG79 obtained using the HTS approach. Table S2.2: The No. of reads for the sequenced amplicons of 96 different *V. vinifera* cultivars of locus VVIb01 obtained using the HTS approach. Table S2.3: The No. of reads for the sequenced amplicons of 96 different *V. vinifera* cultivars of locus VVIn73 obtained using the HTS approach. Table S2.4: The No. of reads for the sequenced amplicons of 96 different *V. vinifera* cultivars of locus VVIp31 obtained using the HTS approach. Table S2.5: The No. of reads for the sequenced amplicons of 96 different *V. vinifera* cultivars of locus VVIq52 obtained using the HTS approach. Table S2.6: The No. of reads for the sequenced amplicons of 96 different *V. vinifera* cultivars of locus VVIv37 obtained using the HTS approach. Table S2.7: The No. of reads for the sequenced amplicons of 96 different *V. vinifera* cultivars of locus VVMD24 obtained using the HTS approach. Table S2.8: The No. of reads for the sequenced amplicons of 96 different *V. vinifera* cultivars of locus VVMD25 obtained using the HTS approach. Table S2.9: The No. of reads for the sequenced amplicons of 96 different *V. vinifera* cultivars of locus VVMD27 obtained using the HTS approach. Table S2.10: The No. of reads for the sequenced amplicons of 96 different *V. vinifera* cultivars of locus VVMD32 obtained using the HTS approach. Table S2.11: The No. of reads for the sequenced amplicons of 96 different *V. vinifera* cultivars of locus VVMD7 obtained using the HTS approach.
